# The truncated isoform of the receptor for hyaluronan-mediated motility (RHAMM^Δ163^) modulates shelterin and telomerase reverse transcriptase transcription affecting telomerase activity

**DOI:** 10.3389/fragi.2025.1604051

**Published:** 2025-06-30

**Authors:** Kaustuv Basu

**Affiliations:** Verspeeten Family Cancer Centre, London Health Sciences Centre, Lawson Health Research Institute, Victoria Hospital, London, ON, Canada

**Keywords:** HMMR, TERT, TRF1, telomerase, shelterin, hyaluronan, aging

## Abstract

**Introduction:**

The receptor for hyaluronan-mediated motility (RHAMM), a centrosomal protein expressing in multiple isoforms, is implicated in telomerase-independent aging. However, its involvement in telomerase regulation is unproven. This study aims to investigate whether RHAMM correlates with telomerase activity in mammalian cells.

**Methods:**

Mouse embryonic fibroblasts expressing or lacking full-length RHAMM (RHAMM^FL^, amino acids 1–794) and the shorter isoform RHAMM^Δ163^ (amino acids 164–794), were explored to examine the effect of RHAMM isoforms on mRNA expression of telomerase reverse transcriptase (TERT) and selective shelterin proteins regulating telomere maintenance.

**Results:**

The preliminary findings revealed that RHAMM regulated *Tert* expression based on its isoforms. RHAMM^Δ163^ enhanced *Tert* mRNA expression and promoted telomerase activity by stimulating sirtuin 1 (*Sirt1*), shelterin proteins *Tpp1*, and *Pot1a* and repressing the telomerase inhibitor *Pinx1* levels. In contrast, RHAMM^FL^ did not have significant effect on TERT expression and telomerase activity. Increasing *Tert* mRNA expression by blocking leucine zipper sequence with function-blocking RHAMM peptide NP-110 in a TERT-deficient mouse model of idiopathic pulmonary fibrosis, alongside suppressing *Tpp1* and *Pot1a* expression in mouse embryonic fibroblasts using ERK1 inhibitor PD98059, highlights the importance of the HATABD domain (amino acids 718–751), which includes leucine zipper and ERK-binding sequences at the C-terminus of mouse RHAMM in regulating telomerase function. Increased telomerase activity raised *Hmmr* expression, suggesting a potential feedback loop between RHAMM and TERT expression.

**Discussion:**

Taken together, this report provides the first evidence that RHAMM^Δ163^ regulates TERT and shelterin expression and telomerase activity in mammalian cells.

## 1 Introduction

Telomeres safeguard chromosome ends with the shelterin complex, which includes six proteins, including telomeric repeat binding factor 1 (TRF1 or TERF1), protection of telomeres protein 1 (POT1), and TPP1 (de Lange, 2005). Each cell cycle leads to telomere attrition due to incomplete synthesis of the G-rich leading strand and the C-rich lagging strand. Telomerase, comprising of telomerase reverse transcriptase (TERT) and an RNA template (TERC) in addition to dyskerin (Dkc1), extends the G-rich strand, while CST-Pola/primase maintains C-rich repeats through fill-in synthesis in coordination with shelterin (Greider and Blackburn, 1985; Cohen et al., 2007; Cai et al., 2024). Notably, telomerase activity and TERT expression in human placenta-derived mesenchymal stem cells are significantly affected when cultured on hyaluronan-coated tissue-culture plate (Wong et al., 2017). Hyaluronan, a key glycosaminoglycan in the extracellular matrix (ECM), plays critical roles in cell migration, proliferation, and differentiation through its receptors, CD44, and receptor for hyaluronan-mediated motility (RHAMM/HMMR) (Heldin et al., 2013), and binding proteins, transcription factors and growth factors (Basu et al., 2025). Although CD44 influences TERT expression (Chung et al., 2013), RHAMM can interact with TERC’s noncoding RNA (Terc-53) but not directly with TERC or TERT (Wu et al., 2025). Notably, neither of these interactions needed hyaluronan (Chung et al., 2013; Wu et al., 2025). While RHAMM is involved in telomerase-independent aging (Wu et al., 2025), its role in telomerase regulation remains unproven.

RHAMM is a unique nuclear protein that expresses negligibly in healthy tissue, lacks an N-terminal signal peptide for conventional export via Golgi/endoplasmic reticulum and is shuttled to the cell surface under stress where it interacts with hyaluronan and CD44 and facilitates cell motility, activating the MAPK/ERK1,2 signaling pathway (Maxwell et al., 2008; Tolg et al., 2006). It binds to actin filaments, microtubules, and centrosomes and helps in spindle formation (Assmann et al., 1999). RHAMM binds hyaluronan via hyaluronan binding domains, HABD1 (amino acids 719–729, mouse) and HABD2 (amino acids 741–750, mouse) in its C-terminus (Figure 2F). HABD1 contains ERK1-binding sequence: ^718^LKQKIKHVVK^727^ which induces interaction with CD44. HABD2 is critical for hyaluronan binding as it contains part of the leucine zipper sequence (^728^LKDENSQLKSEVSKL^742^) which stabilizes the helical hyaluronan binding sequences and is critical for RHAMM’s interaction with targeting protein for XKlp2 (TPX2), and aurora kinase A (AURKA) (Tolg et al., 2010). Combination of both the sequences results in ‘Hyaluronan-Tubulin-AURKA Binding Domain’ (HATABD) (amino acids 718–751, mouse). Notably, Terc-53 binds to Hmmr sequence (amino acids 596–794) which includes ‘HATABD’ domain (Wu et al., 2021).

**FIGURE 1:**
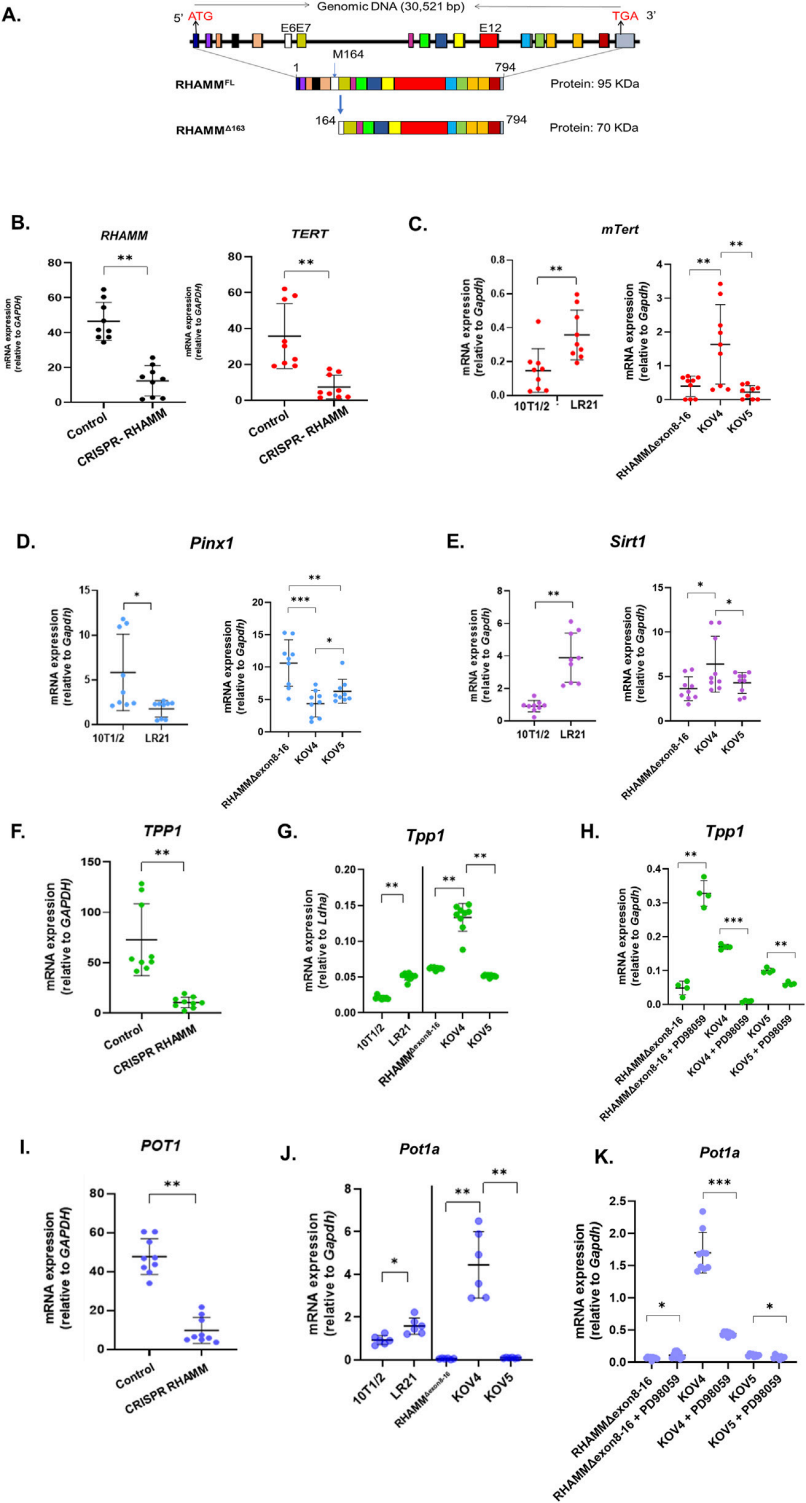
RHAMM^Δ163^ regulates telomerase-mediated telomere elongation. RHAMM is present in multiple isoforms, including RHAMM^FL^ and RHAMM^Δ163^ in mice **(A)**. HMMR was eliminated using CRISPR-Cas9 in MDA-MB-231 cells. RNA was isolated, and qRT-PCR was used to measure the mRNA expression of *TERT, TPP1*, and *POT1*, normalized to *Gapdh*. Data are means ± SD; n = 3 experiments; *p < 0.05, **p < 0.01, ***p < 0.001 by Student’s t-test **(B,F,I)**. RNA was also isolated from 10T1/2, LR21, RHAMM^Δexon8-16^, KOV4, and KOV5 cell lines, with mRNA levels for *mTert*, *Pinx1, Sirt1, Tpp1*, and *Pot1a* measured similarly **(C,D,E,G,J)**. For RHAMM^Δexon8-16^ (KO) and rescued lines (KOV4 and KOV5), treatment with PD98059 at 50 µM for 24 h was followed by qRT-PCR analysis to measure the mRNA expression of *Tpp1*, and *Pot1a*, normalized to *Gapdh*. Results are means ± SD; n = 2 experiments; *p < 0.05, **p < 0.01, ***p < 0.001 **(H,K)**.

RHAMM is expressed as multiple isoforms due to alternative splicing and the use of alternate start codons (Messam et al., 2021). In mice, in addition to the five natural isoforms of RHAMM, a truncated isoform RHAMM^Δ163^ (also referred to as RHAMMv4, amino acids 164–794) was variably detected in 3T3 cells using techniques such as 5′RACE, primer extension, and RT-PCR, corresponding to a protein size of 70–73 kDa (Zhang et al., 1998) (Figure 1A). This isoform is capable of transforming fibroblasts and exhibits oncogenic potential (Hall et al., 1995). Notably, high telomerase activity is common in cancers (Blasco, 2003). The protein expression of the full-length RHAMM (RHAMM^FL^, amino acids 1–794, MW: 95 kDa) and RHAMM^Δ163^ isoforms was confirmed in different cell lines using *in vitro* translation followed by Western Blot analysis (Telmer et al., unpublished). Of note, high telomerase activity is common in cancers (Blasco, 2003). The shorter isoform in mice, RHAMM X1 (MW: 87 kDa), binds more strongly to Terc-53 than RHAMM^FL^ (Wu et al., 2025). Additionally, the isoform RHAMM^Δ163^ can enter the nucleus but RHAMM^FL^ cannot (Tolg et al., 2010). This suggests that the shorter isoform of RHAMM behaves differently from the full-length RHAMM and has distinct functions. Taken together, it was hypothesized that RHAMM^Δ163^, unlike RHAMM^FL^, could be linked to TERT expression through the HATABD domain.

To test the hypothesis, seven cell lines were used. Commercially available 10T1/2, a mouse embryonic fibroblast cell line expressing endogenous RHAMM^FL^ and a small amount of RHAMM^Δ163^ (Tolg et al., 2010), was modified in Dr. Eva Turley’s laboratory at Western University, Canada, to overexpress RHAMM^Δ163^, resulting in LR21 cells (Tolg et al., 2006). The other three murine cell lines were generated from mouse model in E. Turley’s research group. RHAMM consists of 18 exons, in which exon 16 contains the leucine zipper sequence and a microtubule-binding sequence facilitating binding to tubulin, essential for the integrity of the mitotic spindle and centrosome. A RHAMM^−/−^ mouse model was developed by deleting exons 8–16 by homologous recombination, retaining exons 1–7 and 17–18, leading to a fusion between exon 7 and 17, causing a frameshift between exon 7 and the hyaluronan-binding region in exon 18 (Tolg et al., 2003). This deletion allows for the expression of a shorter 920 bp N-terminal mRNA but not the C-terminus and HATABD sequence. From the embryos of these mice, a RHAMM^Δexon8-16^ (KO) cell line was generated, which expresses non-oncogenic truncated N-terminal isoforms of RHAMM (RHAMM^Δexon8-16^), which promoted pancreatic cancer progression in partnership with heterozygous p53 knockout (Tolg et al., 2003; Lin et al., 2021). RHAMM^FL^ and RHAMM^Δ163^ were rescued in the RHAMM^Δexon8-16^ (KO) cell line, labeled KOV5 and KOV4, respectively. Additionally, the human breast cancer MDA-MB-231 cell line, known for its high telomerase activity, was explored, along with RHAMM^−/−^ MDA-MB-231 cells, to investigate the role of RHAMM in telomerase activity.

## 2 Materials and methods

### 2.1 Cell culture

The mouse embryonic fibroblast (MEF) cell lines: 10T1/2 (purchased from ATCC (Manassas, VA)), RHAMM^Δ163^-overexpressing (LR21) cells, RHAMM^Δexon8-16^ (KO), RHAMM^Δexon8-16^ rescued with RHAMM^Δ163^ (KOV4) and RHAMM^Δexon8-16^ rescued with RHAMM^FL^ (KOV5) and the human breast cancer cell lines: MDA-MB-231 wild type and depleted of RHAMM using CRISPR/Cas9 were kind gifts from Dr. E. Turley, Western University, Canada and Dr. J. McCarthy, University of Minnesota. Because of different origin, the analysis was done by comparing 10T1/2 vs. LR21 and by comparing KO vs. KOV4 vs. KOV5. All the cell lines used in the study are telomerase positive. MDA-MB-231 cell line was used because telomerase activity is high in these human cells. The panel of murine cells were used to study the importance of N- and C- terminus of RHAMM and different isoforms of RHAMM. The cells were prepared and cultured to sub-confluency using Dulbecco’s modified Eagle’s medium (DMEM) (DMEM, 4.5 g/L Glucose; ThermoFisher, Cat # 21068028) containing 10% fetal bovine serum (FBS; Sigma Aldrich, cat #F0926), 4 μg/mL insulin, 8 μg/mL transferrin under the standard culture conditions of 37^o^C in a humidified 5% CO_2_ atmosphere as previously described (Tolg et al., 2003). The medium was changed every 2–3 days. The confluent cells were gently washed with sterile Phosphate Buffer Saline (PBS), pH 7.2–7.4, followed by harvested using TrypLE™ Express Enzyme (1X) (Gibco, Cat # 12563011).

### 2.2 Cell treatment

The 10T1/2 fibroblasts were cultured in a complete medium for 24 h, then switched to a serum-starved medium (SSM) with 1% FBS. Cycloastragenol (CAG; Cat # SML1448, Sigma-Aldrich, United States) was added at 3 µM for 6 h, while control cells received DMEM in SSM. After 6 h, removing the medium, the cells were washed twice with cold PBS for qRT-PCR analysis. RHAMM^Δexon8-16^ (KO), KOV4 and KOV5 were treated with PD98059 at 50 µM in SSM for 24 h before qRT-PCR analysis.

### 2.3 Telomeric Repeat Amplification Protocol

The TRAPeze Gel-based Telomerase Detection Kit (Cat #S7700, Millipore Sigma, United States) was used to perform the telomerase repeat amplification protocol (TRAP) assay according to the manufacturer’s instructions. Telomerase adds an AG sequence and telomeric repeats to the 3′end of substrate oligonucleotide (TS), followed by amplification using polymerase chain reaction (PCR) with unlabeled TS and reverse (RP) primers. This produces a ladder of products starting at 50 nucleotides in six-base increments. The PCR conditions were 94°C for 30 s, 59°C for 30 s, and 72°C for 1 min over 30 cycles. The products were analyzed on a 1% agarose gel, including controls for positive telomerase activity, PCR contamination, and a TSR-8 template as provided by the kit. Two independent experiments (n = 2) were conducted to validate the results.

### 2.4 Extracellular hyaluronan

The 10T1/2 fibroblasts were cultured in six-well plates with complete medium for 24 h, followed by serum starvation with 1% FBS for another 24 h. The conditioned medium was collected, and extracellular hyaluronan was quantified using the Hyaluronan Quantikine ELISA Kit (R&D, Cat # DHYAL0) according to the manufacturer’s instructions. In this assay, standards, controls, and samples were added to a microplate pre-coated with recombinant human (rh) aggrecan, allowing hyaluronan to bind to it. After washing to remove unbound substances, enzyme-linked rh aggrecan was added, followed by a substrate solution that developed color proportional to the bound hyaluronan. After stopping color development, the optical density was measured within 30 min at 450 nm. hyaluronan concentration was normalized to 1 μg of RNA extracted from cells, with data presented as means ± standard deviation (SD) from three replicates.

### 2.5 RHAMM functional assay

The significance of the hyaluronan binding and leucine zipper sequences was evaluated using a function-blocking RHAMM peptide, NP-110, in lung tissue from C57BL/6J mice. This tissue was sourced from a previous study (Wu et al., 2021) (kind gifts from Dr. E. Turley, Western University, Canada) in which the animal work was conducted by Stelic MC, Inc. in Tokyo, Japan, with institutional ethics approval and per the Guidelines for Proper Conduct of Animal Experiments (Science Council of Japan). In total, 36 six-week-old female C57BL/6J mice were randomly assigned to one of three groups: (i) the control group receiving 50 µL of 0.9% saline; (ii) the bleomycin (BLM) group receiving 50 µL of 1.5 mg/mL Bleomycin sulfate (Lot #15180, Nippon Kayaku, Japan) for 28 days; and (iii) BLM group receiving 3 mg/kg of NP-110 (sequence: ^644^KLKDENSQLKSEVSK) every fourth day (i.e., day 0, 4, 8, 12, 16, 20, 24) after BLM injections. Mice were subjected to sacrifice on day 28, and lungs were harvested and stored at −80 ± 5^o^C for future use.

### 2.6 RNA extraction and quantitative real time polymerase chain reaction (qRT-PCR)

RNA isolation and qRT-PCR were performed according to established protocols (Tolg et al., 2017). Briefly, TRIZOL reagent (Ambion, United States) was used to isolate total RNA from the cells and lung tissue following the manufacturer’s instructions. Complementary DNA (cDNA) synthesis was conducted using iScript Reverse Transcription Supermix (BioRad, United States). Primers were designed with Primer three software (version 0.4.0) and synthesized by Life Technologies, United States (Supplementary Table S2). PCR reactions were set up with 1 μg of cDNA using the SsoAdvanced Universal SYBR Green Supermix kit (BioRad, United States). The PCR cycling conditions included an initial denaturation step at 95°C for 10 min, followed by 40 cycles of denaturation at 95°C for 30 s, annealing at 60°C for 1 min, and extension at 72°C for 1 min. All samples were analyzed in triplicate. Expression analysis and calculation of relative changes in gene expression were carried out using Stratagene Mx3000Pro software and MS Excel. Mouse gene expression was normalized to the *Gapdh, Ldha,* and *Snrpd3* while human gene expression was normalized to *GAPDH* and *HPRT1.*

### 2.7 *In-silico* analysis

**FIGURE 2:**
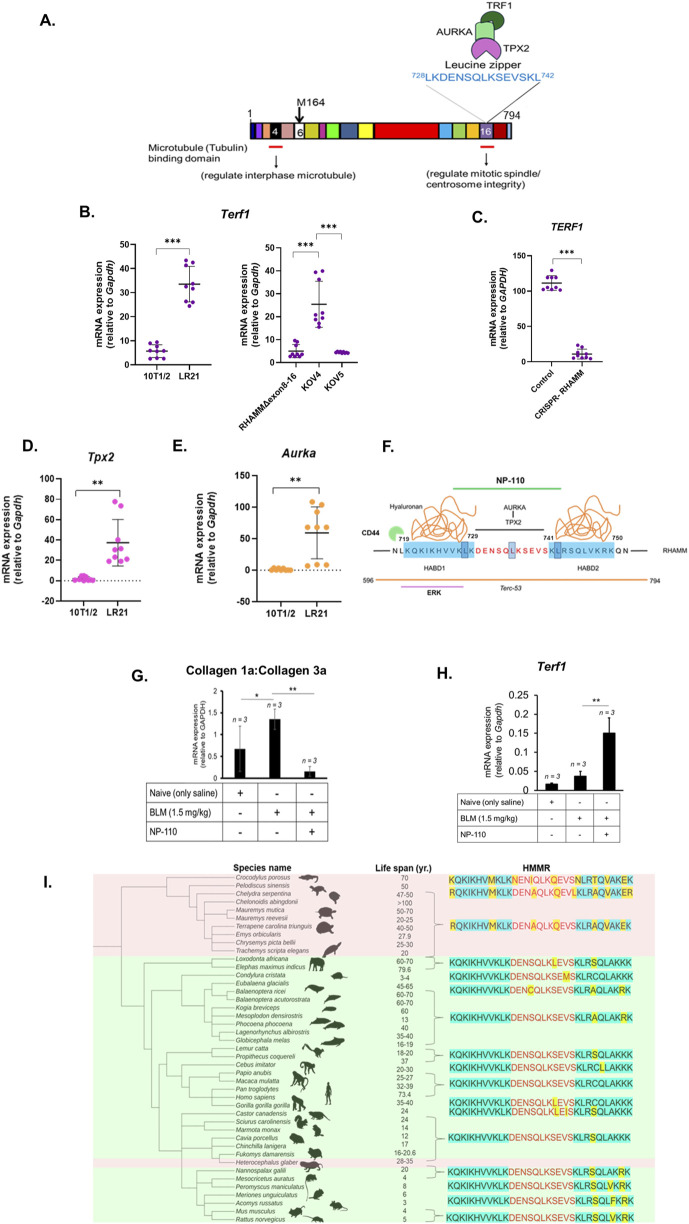
RHAMM regulates TERT expression via shelterin and ‘HATABD’ domain. A cartoon illustrates mouse RHAMM’s interaction with microtubules, TPX2, AURKA, and TRF1. The leucine zipper motif is highlighted **(A)**. qRT-PCR was used to measure the mRNA expression of *Terf1, Aurka*, *Tpx2*, *col1A* and *Col3A*, normalized to *Gapdh*. Data are means ± SD; n = 3 experiments; *p < 0.05, **p < 0.01, ***p < 0.001 by Student’s t-test **(B,D,E,G,H)**. HMMR was eliminated using CRISPR-Cas9 in MDA-MB-231 cells. RNA was isolated, and qRT-PCR was used to measure the mRNA expression of *TERF1* normalized to *Gapdh*. Data are means ± SD; n = 3 experiments; *p < 0.05, **p < 0.01, ***p < 0.001 by Student’s t-test **(C)**. Functional-blocking RHAMM peptide NP-110 (green line) inhibits hyaluronan (orange) binding (blue) and TPX2-AURKA binding domain with leucine zipper sequence (red) in RHAMM. The orange line denotes the Terc-53 binding domain, and the purple line indicates the ERK1-binding sequence **(F)**. Importance of ‘HATABD’ domain of RHAMM in aging across the animal kingdom. Phylogenetic correlation between ectotherm (pink) and endotherm (green) vs. HMMR functional domains (HATABD) with reference to human HMMR sequence. Molecular changes are marked yellow **(I)**.

The Protein sequences of RHAMM from different animals, shown in Figure 2I were sourced from the NCBI Protein database (https://www.ncbi.nlm.nih.gov/protein/). NCBI-BLAST (https://blast.ncbi.nlm.nih.gov/Blast.cgi) checked the homology of the protein sequences of the ‘HATABD’ domain in RHAMM across the phylogenetic tree in the animal kingdom. Phylogenetic tree was drawn with iTOL (Letunic and Bork, 2024), and silhouettes are available on phylopic.org. We analyzed mRNA expression data of HMMR and hTERT using clinical datasets of ten different types of cancer retrieved from the TCGA database using cBioPortal (Cerami et al., 2012).

### 2.8 Statistical analysis

The difference between experimental groups of two-sample unequal variance was analyzed using Student’s t-test, with *p < 0.05, **p < 0.01, ***p < 0.001 being considered significant. The experiments were performed at least two to three times independently and represented as bar diagrams or dot plots using GraphPad Prism, ver. 10.3.1 (URL: https://app.graphpad.com).

## 3 Results

### 3.1 RHAMM^Δ163^ enhances, but RHAMM^FL^ does not affect TERT mRNA expression and telomerase activity

The mRNA expression levels of RHAMM and TERT are positively correlated (p < 0.05) across ten types of human cancer and cancer cell lines, obtained from the TCGA Pan-Cancer Atlas database (Supplementary Table S1). To investigate this correlation, RHAMM was eliminated in the MDA-MB-231 cancer cell line using CRISPR/Cas9 (kind gift from E. Turley), leading to a significant 3-fold decrease (p < 0.01) in TERT mRNA expression (Figure 1B). A TRAP assay revealed that RHAMM-depleted cells had lower telomerase activity than those expressing RHAMM^Δ163^ (Supplementary Figure S1A; original gel image shown in Supplementary Figure S3). Because RHAMM is expressed in multiple isoforms exhibiting different functions, further investigation revealed that RHAMM^Δ163^, significantly increased TERT mRNA levels (p < 0.01; Figure 1C). However, TERT mRNA level remained unchanged in RHAMM^Δexon8-16^ cells rescued with RHAMM^FL^ or depleted of RHAMM^FL^ and RHAMM^Δ163^, suggesting that RHAMM^FL^ has no effect on TERT mRNA expression (Figure 1C). Furthermore, this result underscores that RHAMM C-terminus is crucial for TERT expression, but not the N-terminus. Similar patterns were observed for mRNA expression of dyskerin (Dkc1), which is critical for telomerase biogenesis (Cohen et al., 2007) (Supplementary Figure S1B). The TRAP assay confirmed that RHAMM^Δexon8-16^ cells expressing RHAMM^Δ163^ affected telomerase activity significantly compared to those with RHAMM^Δexon8-16^ cells lacking RHAMM^Δ163^ (Supplementary Figure S1A).

### 3.2 RHAMM^Δ163^ suppresses PINX1 expression and enhances TPP1-POT1 complex

Telomere length is controlled through multiple mechanisms involving telomerase regulators and shelterin proteins (de Lange, 2005). PINX1 directly binds to TERT and inhibits telomerase catalytic activity endogenously (Zhou and Lu, 2001). Conversely, the NAD-dependent deacetylase sirtuin 1 (SIRT1) interacts with telomeric repeats and positively regulates telomere elongation (Palacios et al., 2010). SIRT1 directly interacts with TERT and regulates its nuclear localization and stability (Lee et al., 2024). In contrast, its abrogation results in increased telomere erosion. SIRT1 acts upstream of TPP1 and interacts with it (Lee et al., 2024) to regulate telomere function and cellular senescence. A reduction in TPP1 levels leads to telomere attrition and cellular senescence associated with SIRT1 (Ahmad et al., 2017). TPP1 forms complex with POT1 which is essential for recruiting telomerase to telomeres (Sekne et al., 2022). TPP1 does not bind to telomeric DNA but interacts with the telomerase essential N-terminal (TEN) domain, which is critical for the TTAGGG repeat addition processivity of telomerase and its recruitment to telomeres, while POT1 flexibly binds both telomeric DNA and telomerase. Together, TPP1 and POT1 enhance telomerase processivity by minimizing DNA dissociation during repeat synthesis, while TPP1-POT1 depletion impairs processive DNA synthesis. Hence, the TPP1-POT1 complex is vital for telomerase processivity (Sekne et al., 2022). This complex protects telomeres (Kibe et al., 2010).

The qRT-PCR analysis showed that RHAMM^Δ163^ significantly reduced the negative regulator of TERT, *Pinx1* mRNA levels while RHAMM^Δexon8-16^ depleted of C-terminus RHAMM and RHAMM^Δexon8-16^ rescued with RHAMM^FL^ increased *Pinx1* expression (Figure 1D). *Pinx1* expression was higher in C-terminus RHAMM-depleted cells compared to RHAMM^FL^-expressing cells (p < 0.01). It could be possible that the N-terminus of RHAMM might enhance *Pinx1* expression, while the C-terminus RHAMM represses it, which remains elusive and warrants further investigation. Thus, RHAMM^Δ163^ may potentially activate TERT expression and lengthen telomeres, while RHAMM^FL^ is likely to inactivate TERT and shorten telomeres. Furthermore, RHAMM^Δ163^ increased the mRNA levels of *Sirt1* (positive regulator of TERT) while neither RHAMM^Δexon8-16^ depleted of C-terminus RHAMM, nor RHAMM^Δexon8-16^ rescued with RHAMM^FL^ had any effect (Figure 1E). This finding indicates that most likely, the N-terminus of RHAMM suppresses *Sirt1* expression, while the C-terminus RHAMM elevates it. Eliminating RHAMM reduced TPP1 and POT1 mRNA expression in MDA-MB-231 (Figures 1F,I), indicating that RHAMM may regulate TPP1-POT1 complex. Further investigation revealed that RHAMM^Δ163^ increased the mRNA levels of *Tpp1* and *Pot1a*, while RHAMM^FL^ exerted no effect on these gene expression (Figure 1G). Taken together, these results show that RHAMM^Δ163^ may promote telomerase activity by increasing SIRT1 and TPP1 expression and suppressing PINX1.

To further understand how RHAMM controls Tpp1 and Pot1a expression, RHAMM^Δexon8-16^ (KO), RHAMM^Δexon8-16^ rescued with RHAMM^Δ163^ (KOV4) and RHAMM^Δexon8-16^ rescued with RHAMM^FL^ (KOV5) were treated with PD98059 (50 µM), an MEK/ERK pathway inhibitor. As previously mentioned, RHAMM binds directly to ERK1 and indirectly to ERK2 and MEK through the sequence ^718^LKQKIKHVVK^727^ at the C-terminus of RHAMM (mouse) (Tolg et al., 2010). This binding is vital for microtubule dynamics and hyaluronan- and CD44-mediated cell motility. The results show that PD98059 significantly decreased *Tpp1* (Figure 1H) and *Pot1a* mRNA expression (Figure 1K) in KOV4 and KOV5 cells, while RHAMM^Δexon8-16^ (KO) which lacks ERK1-binding domain (amino acids 718–727, mouse) promoted decreased *Tpp1* and *Pot1a* levels*.* This result suggests that RHAMM^Δ163^ may regulate TPP1 and POT1 expression through the ERK-mediated signaling pathway, possibly via the ERK1-binding domain, which needs further validation. Notably, RHAMM, CD44, and ERK1,2 form a complex that is required for activating ERK1,2 (Tolg et al., 2006). qRT-PCR analysis revealed a significant increase in *Cd44* mRNA expression in RHAMM^Δexon8-16^ rescued with RHAMM^Δ163^ (KOV4) compared to RHAMM^Δexon8-16^, with and without RHAMM^FL^ (Supplementary Figure S2A).

### 3.3 RHAMM^Δ163^ enhances TRF1 mRNA expression

During mitosis, chromosome segregation relies on the organization of polymeric tubulin into bipolar spindles, with RHAMM decorating these spindles. As demonstrated in Figure 2A, the interaction of RHAMM with the cytoskeleton and centrosomes through TPX2 and AURKA is crucial for mitotic spindle assembly (Chen et al., 2014). Excessive AURKA upregulation disrupts normal mitosis, causing multinucleated cells and centrosome amplification. This is linked to TRF1, which stabilizes microtubule-kinetochore connections essential for chromosome segregation. AURKA phosphorylates TRF1, and excessive phosphorylation can lead to mitotic abnormalities (Ohishi et al., 2010). Notably, TRF1 is linked to TPP1 and POT1 via TIN2 and utilizes them to prevent ATR kinase during telomere replication and suppress sister telomere associations (Zimmermann et al., 2014).

RHAMM^Δ163^ increased *Trf1* levels significantly (Figure 2B). In contrast, RHAMM^Δexon8-16^, with and without RHAMM^FL^ did not affect *Trf1* expression (Figure 2B). The elimination of RHAMM decreased *TRF1* mRNA expression in MDA-MB-231 cells indicating that RHAMM positively regulates TRF1 (Figure 2C). Ectopic expression of RHAMM^Δ163^ elevated *Tpx2* (Figure 2D) and *Aurka* mRNA levels (Figure 2E), provokes to hypothesize that RHAMM^Δ163^ may regulate TRF1 transcription through TPX2-AURKA-mediated pathways, which needs further investigation. Notably, TRF1 interacts with BubR1, Nek2, Mad1, and Mad2 in the mitotic spindle checkpoint, playing a role in spindle formation (Muñoz et al., 2009; Prime and Markie, 2005). It is not clear if RHAMM and TRF1 act together in mitotic spindle regulation. However, evaluation of unpublished microarray analysis of 10T1/2 and LR21 cells (Tolg et al., 2012) indicates that RHAMM^Δ163^ increases mRNA expression of Mad1l1, Mad2l1, and Nek2 (Table 1). This suggests that RHAMM^Δ163^ affects TRF1-partners in mitotic spindle checkpoint (Figure 2E). Furthermore, RHAMM^Δ163^ increases *Rtel1* and *Blm* mRNA levels (Table 1). Notably TERF1 recruits these essential helicases, which resolve replication-associated issues and suppress the fragile-telomere phenotype. Additionally, RHAMM^Δ163^ represses the mRNA level of telomerase-associated protein 1 (Tep1) (Table 1). Taken together, this pilot study suggests that RHAMM^Δ163^ affects TERF1 expression, which may have paramount importance in telomere elongation and mitotic spindle regulation.

**TABLE 1:** Gene expression related to telomere function and mitotic spindle integrity in 10T1/2 mouse embryonic fibroblasts overexpressing RHAMM^Δ1^.^63^.

Gene	Description	Ratio (10T1/2:LR21)	Fold change (10T1/2 vs. LR21)	Expression
*Terf1/Trf1*	Telomeric repeat binding factor 1	0.49	−2.33	Promotion
*Aurka*	Aurora kinase A	0.43	−2.67	Promotion
*Rtel1*	Regulator of telomere elongation helicase 1	0.76	−1.54	Promotion
*Blm*	Bloom syndrome, RecQ helicase-like	0.62	−1.60	Promotion
*Tep1*	Telomerase associated protein 1	1.71	1.71	Repression
*Mad2l1*	MAD2 mitotic arrest deficient-like 1 (yeast)	0.40	−2.47	Promotion
*Nek2*	NIMA (never in mitosis gene a)-related expressed kinase 2	0.44	−2.27	Promotion
*Mad1l1*	Mitotic arrest deficient 1-like 1	0.62	−1.60	Promotion

Note: Unpublished data from (Tolg et al., 2012). LR21 = 10T1/2 cells overexpressing RHAMM^Δ163^.

### 3.4 Importance of HATABD sequence of RHAMM for TERT expression

To investigate the importance of the ‘HATABD’ sequence of RHAMM on TERT mRNA expression, a mouse model with idiopathic pulmonary fibrosis (IPF) was chosen as IPF is characterized by TRF1 deletion or Tert deficiency, especially following a bleomycin (BLM) challenge (Povedano et al., 2015). HAS2 dysregulation in IPF (Li et al., 2016) and the effects of RHAMM antibodies and peptide mimetics in reducing macrophage recruitment and early fibrosis (Zaman et al., 2005) highlight the connection between RHAMM, hyaluronan, and IPF. In a prior study, the function-blocking RHAMM peptide, NP-110, which sterically disrupts RHAMM’s association with hyaluronan, TPX2, and AURKA (Figure 2F), was injected into BLM-treated mice, reducing fibrosis and increasing antifibrotic adipokines in skin (Wu et al., 2021). The current study analyzed the retained lung tissues from the previous study (a generous gift from Dr. E. Turley, Western University), revealing that NP-110 administration decreased the collagen 1a to collagen 3a ratio (Figure 2G) and elevated mTert mRNA expression almost three-fold (Figure 2H). These findings emphasize that blocking the interaction of RHAMM with hyaluronan, TPX2, tubulin, and AURKA via HATABD sequence may control IPF by regulating TERT expression.

The HATABD domain’s significance in regulating TERT expression by RHAMM prompted to screen the sequence across the animal kingdom *in silico* to understand if this sequence could be linked to longevity and telomere maintenance. The animals were selected based on their lifespan. The protein sequence homology of HATABD domain was examined in selected long-lived and short-lived species, using human RHAMM (HMMR) as a reference, with key amino acid changes highlighted in yellow (Figure 2I). The LKQKIKHVVK sequence in HABD1 is crucial for binding ERK1 (Tolg et al., 2010). Long-lived ectothermic reptiles showed two modifications (K636R and V644M), except crocodiles (demonstrating only V644M). These changes may influence RHAMM-ERK binding, although their connection to aging and telomere function is unclear. Stable HABD1 sequences were found in endothermic animals and long-lived naked mole rats. Additionally, the leucine zipper sequence (LKDENSQLKSEVSKL) contains three serine residues that were modified in long-lived ectothermic reptiles but not in short-lived species (Figure 2I). In-depth functional study is required to investigate the importance of these modifications on RHAMM interactions. Ectothermic tetrapods like crocodiles, turtles, and salamanders tend to age more slowly and have longer lifespans than birds and mammals (Reinke et al., 2022). This bioinformatic study prompts to hypothesize that amino acid substitutions in the ‘HATABD’ domain of RHAMM, under selection pressure, may relate to telomere maintenance and longevity, which warrants experimental validation.

### 3.5 Telomerase activity affects RHAMM mRNA expression

**FIGURE 3:**
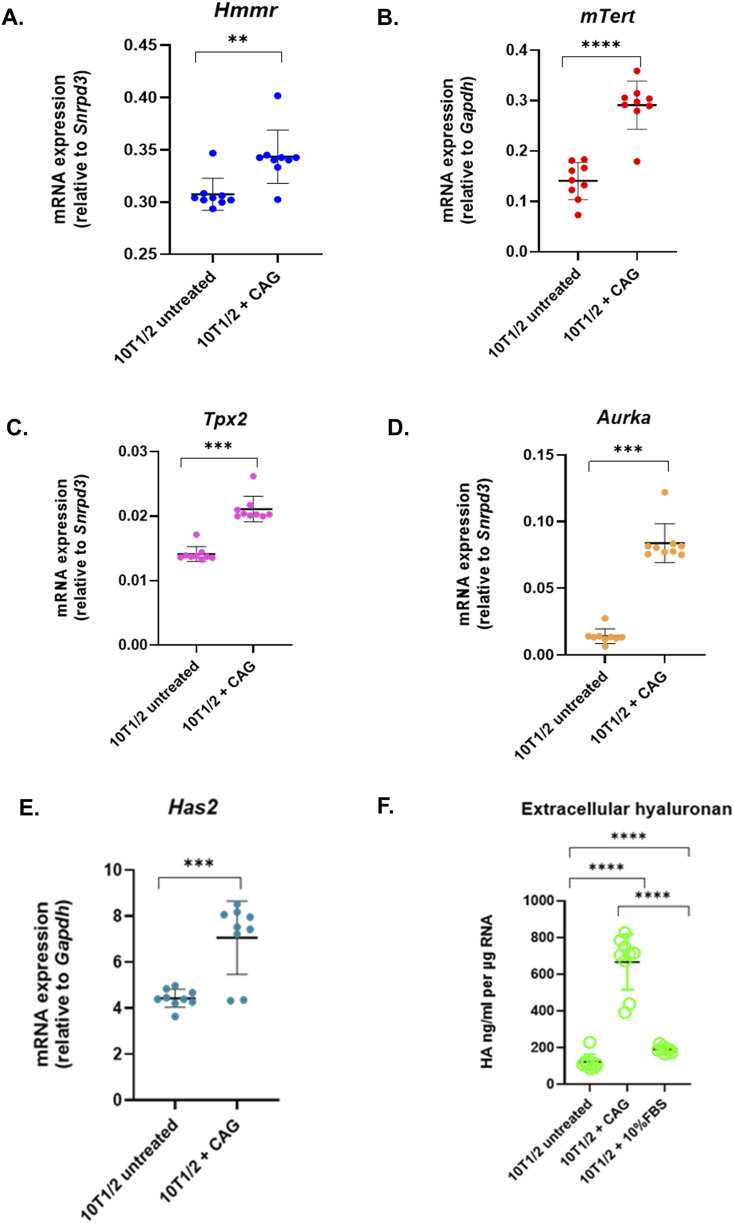
Telomerase activity affects RHAMM expression. Cycloastragenol (3 µM) was added to 10T1/2 fibroblasts for 6 h, followed by RNA isolation and qRT-PCR analysis to measure the mRNA expression of *Hmmr, mTert, Tpx2*, *Aurka*, and *Has2,* normalized to *Gapdh* or *Snrpd3*. Data are means ± SD; n = 3 experiments; *p < 0.05, **p < 0.01, ***p < 0.001 by Student’s t-test **(A–E)**. The extracellular hyaluronan concentration was analyzed by hyaluronan-based ELISA assay. The hyaluronan level was normalized by total RNA concentration **(F)**.

To examine the impact of telomerase activity and TERT expression on RHAMM, 10T1/2 fibroblasts were treated with telomerase activator, cycloastragenol (CAG), a triterpenoid saponin from *Astragalus membranaceus* (Yang et al., 2023). This treatment significantly increased mRNA levels of Hmmr and Tert (p < 0.05) (Figures 3A,B) and enhanced mRNA expression of *Tpx2* and *Aurka* (Figures 3C,D). It enhanced *Has2* mRNA expression (Figure 3E), resulting in a nearly four-fold increase in hyaluronan synthesis in treated cells (600 ng/mL) compared to untreated cells (150 ng/mL, p < 0.05) (Figure 3fF. These findings suggest that telomerase activation and TERT upregulation may significantly enhance *Hmmr* expression and indicate a potential feedback loop with TERT, as noted for CD44 (Chung et al., 2013).

## 4 Discussion

This pilot study reveals, for the first time, that RHAMM regulates telomerase expression and activity, with different isoforms having distinct roles. The C-terminus of RHAMM is critical for TERT expression via the HATABD domain, which contains the leucine zipper (LKDENSQLKSEVSKL) and ERK1-binding sequences (LKQKIKHVVK). The study demonstrates that RHAMM^Δ163^ affects the expression of protein components of telomerase, i.e., TERT and dyskerin, as well as the expression of shelterin proteins TRF1, TPP1, POT1, and TRF2IP (Supplementary Figure S2B) and telomerase activity, supporting TERT-mediated telomere maintenance. Notably, POT1/TPP1 protects telomeres (Kibe et al., 2010) and regulates the synthesis of the C-rich lagging strand through their interaction with CST-Polα/primase (Cai et al., 2024). The possibility of RHAMM^Δ163^’s contribution to these processes cannot be ruled out. In contrast, RHAMM^FL^ does not affect the mRNA expression of TERT and shelterin proteins. Based on previous findings (Messam et al., 2021), it is speculated that nuclear localization of RHAMM^Δ163^ but not RHAMM^FL^ may play a significant role in triggering TERT mRNA expression. However, the possibility of a potential telomerase inhibitor domain (TID) at the N-terminus of RHAMM (amino acids 1–163, mouse) cannot be ruled out. Because the RHAMM^Δexon8-16^ isoform is non-oncogenic (Lin et al., 2021) and originated from the N-terminus, it is unlikely that it will affect TERT expression and have an additive effect on RHAMM^Δ163^. However, further investigation may be required to understand the role of RHAMM^Δexon8-16^ in telomerase regulation. An *in vivo* assay with the function-blocking RHAMM peptide NP-110 highlights the importance of RHAMM’s interaction with hyaluronan, tubulin, ERK1, and aurora kinase A via HATABD domain to regulate TERT expression. NP-110 peptide may offer a therapeutic approach to modulate telomerase function and control idiopathic pulmonary fibrosis which needs further validation in larger cohorts of mice. A limitation of the study is that it exclusively investigated mRNA expression through qRT-PCR analysis, without addressing protein analysis. Furthermore, it is unclear whether telomerase affects RHAMM expression through TERT activity or an off-target mechanism. Additional experiments are necessary to address this question. However, this study, along with Wu et al. (2021), identifies RHAMM as a key telomere-associated protein involved in both telomerase-dependent and independent aging, suggesting it as a promising biomarker for aging.

## Data Availability

The original contributions presented in the study are included in the article/Supplementary Material, further inquiries can be directed to the corresponding author.
