# Supplementary material for: The truncated isoform of the receptor for hyaluronan-mediated motility (RHAMMΔ163) modulates shelterin and telomerase reverse transcriptase transcription affecting telomerase activity
Source: Front Aging. 2025 Jun 30;6:1604051. doi: 10.3389/fragi.2025.1604051 (PMC12256479; doi:10.3389/fragi.2025.1604051)
Supplement: Supplementary file 1 [file Table1.docx]

| **Cancer type** | **Spearman co-efficient** | **p-value** | **No. of samples (n)** |
| --- | --- | --- | --- |
| Cancer cell lines* | 0.12 | 3.106e-5 | n=1739 |
| Breast invasive carcinoma | 0.07 | 8.65e-24 | n=1108 |
| Bladder urothelial carcinoma | 0.21 | 2.185e-5 | n=411 |
| Uveal melanoma | 0.42 | 1.144e-4 | n=80 |
| Kidney renal papillary cell carcinoma | 0.44 | 4.26e-4 | n=283 |
| Liver hepatocellular carcinoma | 0.42 | 5.74e-17 | n=372 |
| Lung adenocarcinoma | 0.29 | 1.23e-11 | n=566 |
| Acute myeloid leukemia | 0.20 | 0.01 | n=165 |
| Ovarian serous cystadenocarcinoma | 0.19 | 1.203e-3 | n=585 |
| Prostate adenocarcinoma | 0.15 | 9.847e-4 | n=494 |
| Skin cutaneous melanoma | 0.24 | 1.95e-7 | n=442 |

**Supplementary Table S1.** Positive correlation between RHAMM and TERT mRNA expression in cancers

**Source:** * Broad Institute, 2019; Rest - TCGA, Pan Cancer Atlas

**Supplementary Table S2.** The list of primers for qRT-PCR analysis

| **Gene** | **Forward primer** | **Reverse primer** |
| --- | --- | --- |
| Mouse | | |
| *Hmmr* | CCTTGCTTGCTTCGGCTAAAA | CTGCTGCATTGAGCTTTGCT |
| *Has2* | TCGCAACACGTAACGCAAT | ACTTCTCTTTTTCCACCCCATTT |
| *Aurka* | CTGGATGCTGCAAACGGATAG | CGAAGGGAACAGTGGTCTTAACA |
| *Tpx2* | GATGCCCCCACCGACTTTATC | CTTGTTCTCCAAGTTGGCCTT |
| *mTert* | GCACTTTGGTTGCCCAATG | GCACGTTTCTCTCGTTGCG |
| *CD44s* | AAGACATCTACCCCAGCAAC | CCAAGATGATCAGCCATTCTGG |
| *mTrf1* | CATGGACTACACAGACTTACAGC | TTCCAAGGGTGTAATACGCTC |
| *Pinx1* | CAGAAACACCGCGTGGAGTAA | TGAGCAATCCAGTTGTCTTCATT |
| *Sirt1* | GCTGACGACTTCGACGACG | TCGGTCAACAGGAGGTTGTCT |
| *Tpp1* | GAGTCTCACTTTTGCGCTGAA | CTCCAGGGTTAGGTACTTTCCA |
| *Pot1a* | TTGGTTTCAACAGCTCCCTATAC | GGAGGGCTTCATAGTTTCCACT |
| *Dkc1* | AAAGACCGGAAGCCATTACAAG | GCCACTGAGAAGTGTCTAATTGA |
| *Gapdh* | AGGTCGGTGTGAACGGATTTG | TGTAGACCATGTAGTTGAGGTCA |
| *Col1a* | CCTCAGGGTATTGCTGGACAAC | CAGAAGGACCTTGTTTGCCAGG |
| *Col3a* | CTGTAACATGGAAACTGGGGAAA | CCATAGCTGAACTGAAAACCACC |
| *TERF2IP* | TGCCTTGTGGAAAGCGATG | TGTTCTGTGGCTCTCCGCTAT |
| *Snrpd3* | ATTGGTGTGCCGATTAAAGTCT | CTGCTTCAATGAGCTTCCCTC |
| *Ldha* | TGTCTCCAGCAAAGACTACTGT | GACTGTACTTGACAATGTTGGGA |
| Human | | |
| *hTERT* | AAATGCGGCCCCTGTTTCT | CAGTGCGTCTTGAGGAGCA |
| *TPP1* | CCTCCACACGGTGCAAAAATG | CTCTGCTTGTCGGATGCTCAG |
| *HMMR* | ATGATGGCTAAGCAAGAAGGC | TTTCCCTTGAGACTCTTCGAGA |
| *POT1* | AGCCTTACGTGTTTGGGCATC | GCATTGGCTGAACATCACACAA |
| *GAPDH* | GGAGCGAGATCCCTCCAAAAT | GGCTGTTGTCATACTTCTCATGG |
| *HPRT1* | CCTGGCGTCGTGATTAGTGAT | AGACGTTCAGTCCTGTCCATAA |
