# Supplementary figures and images for: The truncated isoform of the receptor for hyaluronan-mediated motility (RHAMMΔ163) modulates shelterin and telomerase reverse transcriptase transcription affecting telomerase activity
Source: Front Aging. 2025 Jun 30;6:1604051. doi: 10.3389/fragi.2025.1604051 (PMC12256479; doi:10.3389/fragi.2025.1604051)

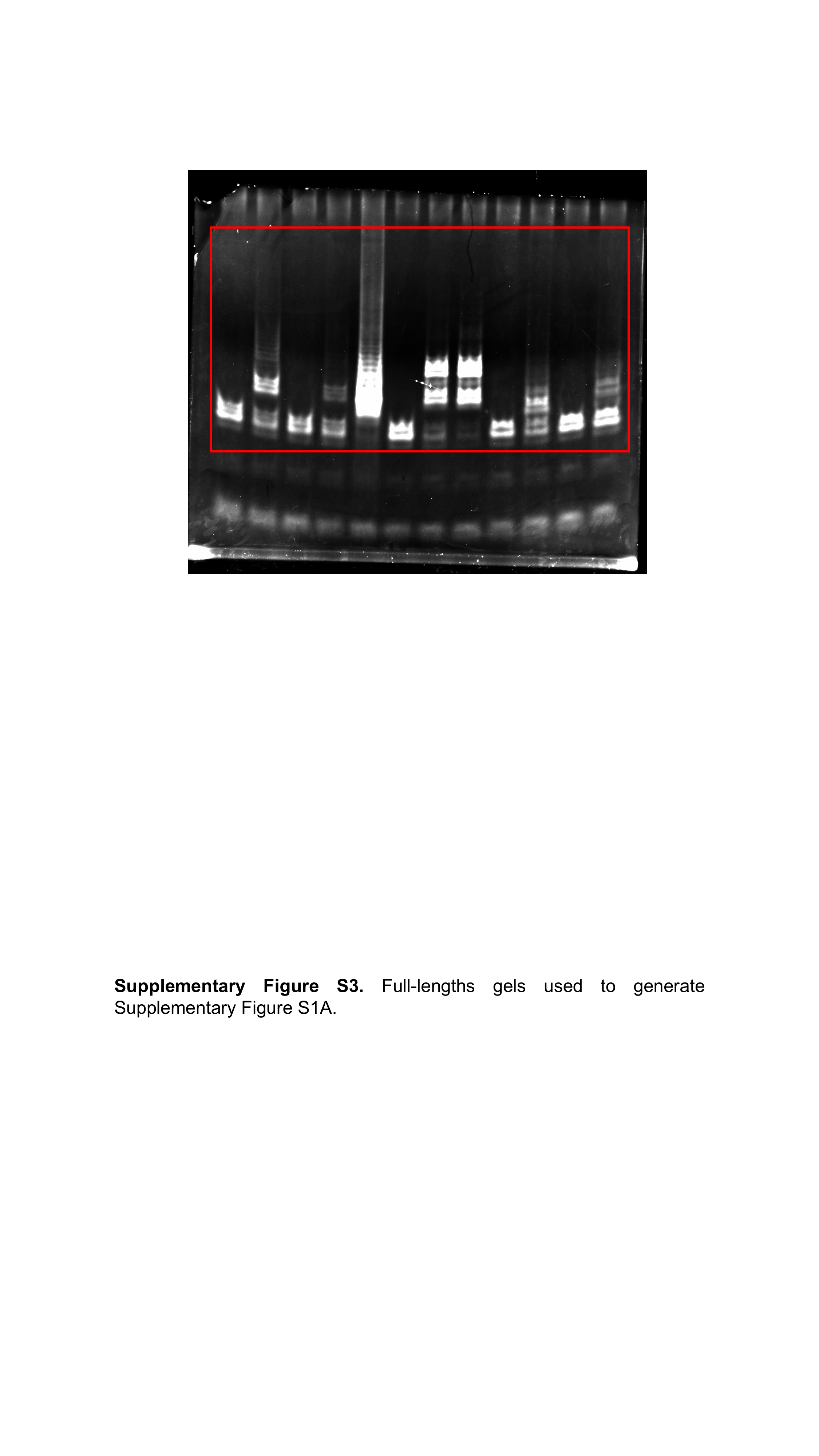

Supplement: Supplementary file 2 [file Image3.tif]

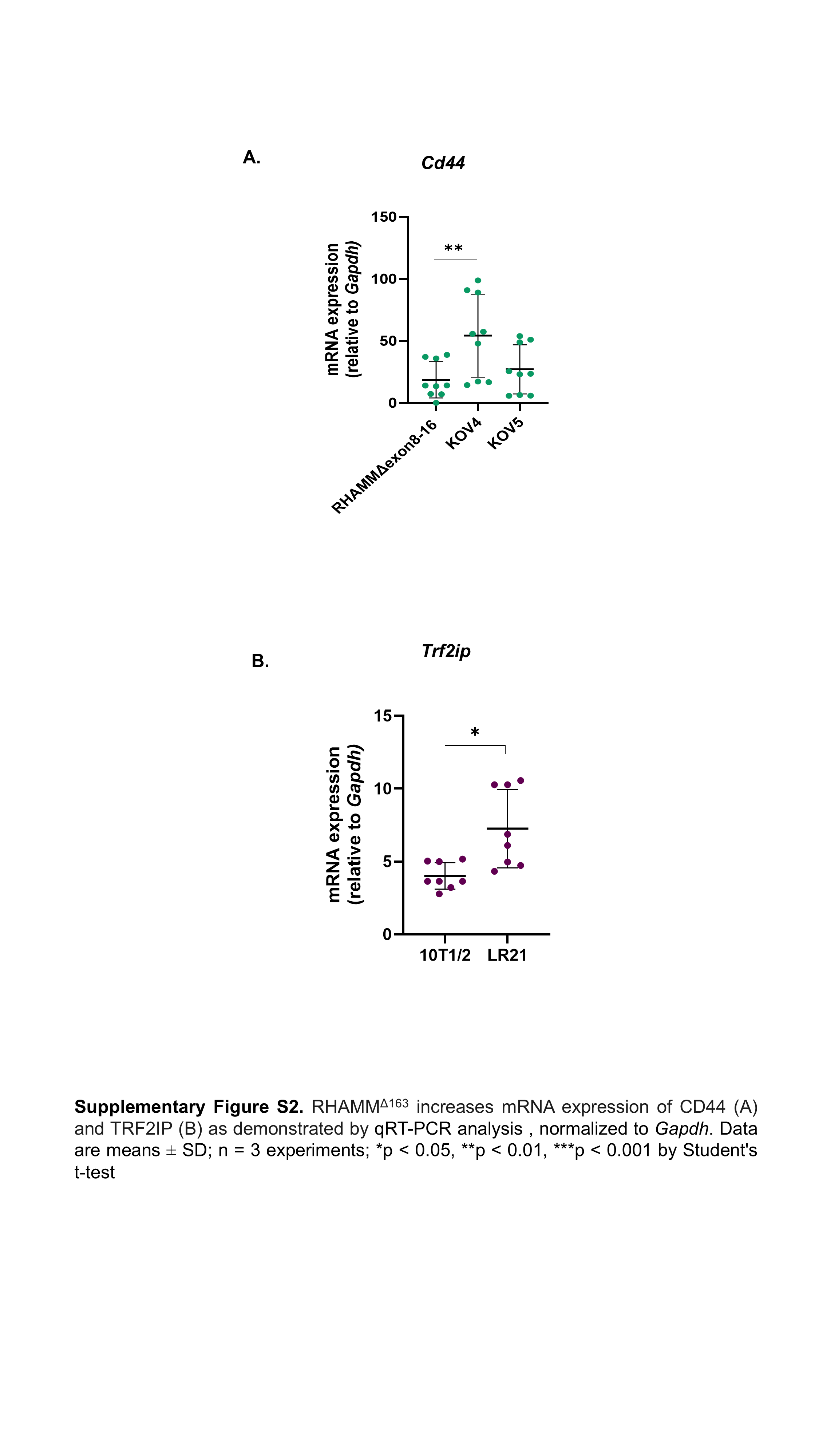

Supplement: Supplementary file 3 [file Image2.tif]

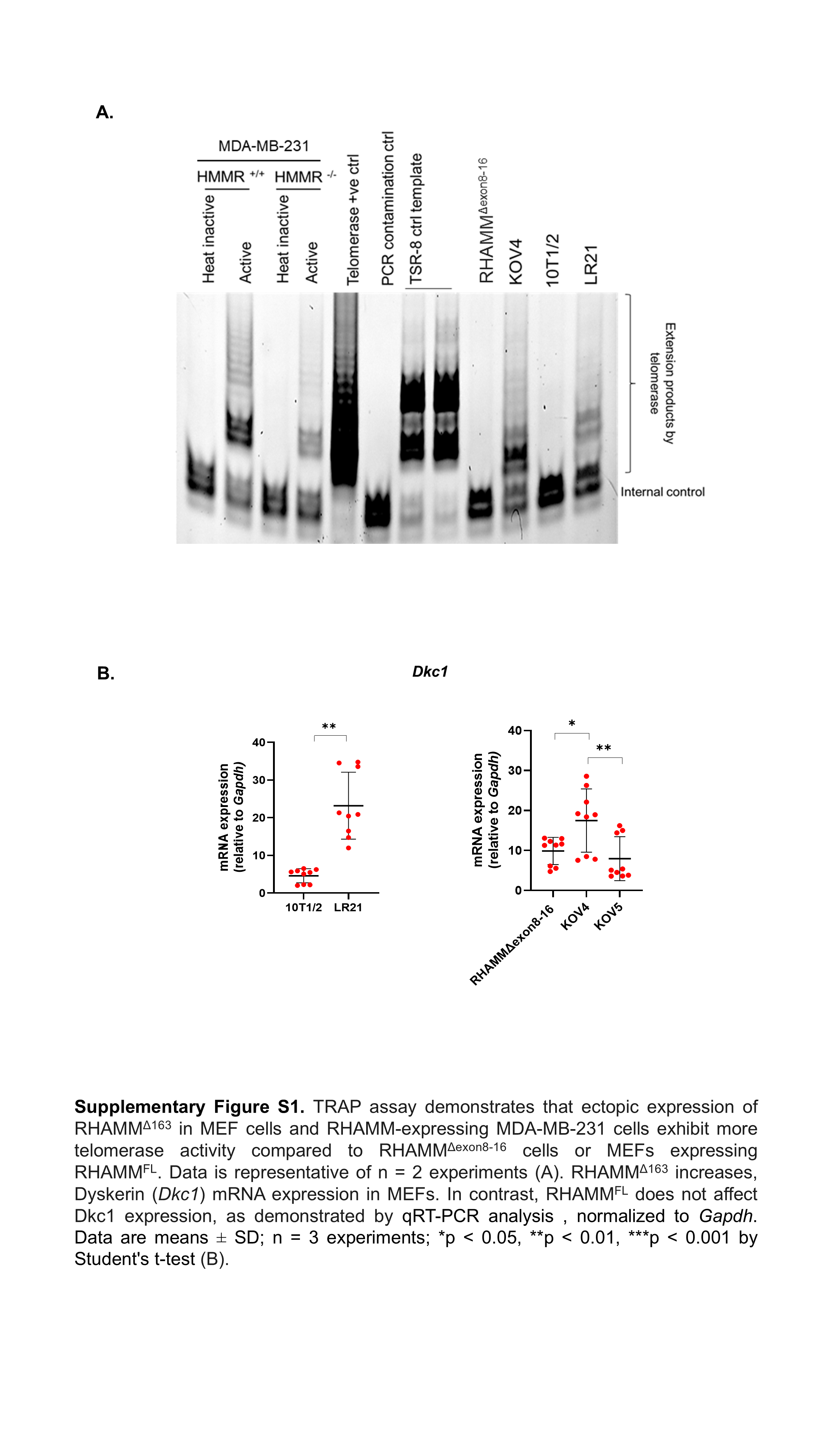

Supplement: Supplementary file 4 [file Image1.tif]
